# Single-cell analysis of [Ca^2+^]_i_ signalling in sub-fertile men: characteristics and relation to fertilization outcome

**DOI:** 10.1093/humrep/dey096

**Published:** 2018-04-25

**Authors:** Mark C Kelly, Sean G Brown, Sarah M Costello, Mythili Ramalingam, Ellen Drew, Stephen J Publicover, Christopher L R Barratt, Sarah Martins Da Silva

**Affiliations:** 1 Reproductive and Developmental Biology, School of Medicine, Ninewells Hospital and Medical School, University of Dundee, Dundee DD19SY, UK; 2 School of Science, Engineering & Technology, Abertay University, Dundee DD11HG, UK; 3 School of Biosciences, The University of Birmingham, Birmingham B15 2TT, UK; 4 Assisted Conception Unit, Ninewells Hospital, Dundee DD19SY, UK

**Keywords:** calcium signalling, CatSper channel, spermatozoa, subfertility, progesterone

## Abstract

**STUDY QUESTION:**

What are the characteristics of progesterone-induced (CatSper-mediated) single cell [Ca^2+^]_i_ signals in spermatozoa from sub-fertile men and how do they relate to fertilizing ability?

**SUMMARY ANSWER:**

Single cell analysis of progesterone-induced (CatSper-mediated) [Ca^2+^]_i_ showed that reduced progesterone-sensitivity is a common feature of sperm from sub-fertile patients and is correlated with fertilization rate.

**WHAT IS KNOWN ALREADY:**

Stimulation with progesterone is a widely used method for assessing [Ca^2+^]_i_ mobilization by activation of CatSper in human spermatozoa. Although data are limited, sperm population studies have indicated an association of poor [Ca^2+^]_i_ response to progesterone with reduced fertilization ability.

**STUDY DESIGN, SIZE, DURATION:**

This was a cohort study using semen samples from 21 donors and 101 patients attending the assisted conception unit at Ninewells Hospital Dundee who were undergoing ART treatment. Patients were recruited from January 2016 to June 2017.

**PARTICIPANTS/MATERIALS, SETTING, METHODS:**

Semen donors and patients were recruited in accordance with local ethics approval (13/ES/0091) from the East of Scotland Research Ethics Service (EoSRES) REC1. [Ca^2+^]_i_ responses were examined by single cell imaging and motility parameters assessed by computer-assisted sperm analysis (CASA).

**MAIN RESULTS AND THE ROLE OF CHANCE:**

For analysis, patient samples were divided into three groups IVF(+ve) (successful fertilization; 62 samples), IVF-FF (failed fertilization; eight samples) and ICSI (21 samples). A further 10 IVF samples showed large, spontaneous [Ca^2+^]_i_ oscillations and responses to progesterone could not be analysed. All patient samples loaded with the [Ca^2+^]_i_-indicator fluo4 responded to progesterone stimulation with a biphasic increase in fluorescence (transient followed by plateau) which resembled that seen in progesterone-stimulated donor samples. The mean normalized response (progesterone-induced increase in fluorescence normalized to resting level) was significantly smaller in IVF-FF and ICSI patient groups than in donors. All samples were further analysed by plotting, for each cell, the relationship between resting fluorescence intensity and the progesterone-induced fluorescence increment. In donor samples these plots overlaid closely and had a gradient of ≈ 2 and plots for most IVF(+ve) samples closely resembled the donor distribution. However, in a subset (≈ 10%) of IVF(+ve) samples, 3/8 IVF-FF samples and one-third of ICSI samples the gradient of the plot was significantly lower, indicating that the response to progesterone of the cells in these samples was abnormally small. Examination of the relationship between gradient (regression coefficient of the plot) in IVF samples and fertilization rate showed a positive correlation. In IVF-FF and ICSI groups, the proportion of cells in which a response to progesterone could be detected was significantly lower than in donors and IVF (+ve) patients. Approximately 20% of cells in donor, IVF(+ve) and ICSI samples generated [Ca^2+^]_i_ oscillations when challenged with progesterone but in IVF-FF samples only ≈ 10% of cells generated oscillations and there was a significantly greater proportion of samples where no oscillations were observed. Levels of hyperactivated motility were lower in IVF(+ve) and IVF-FF groups compared to controls, IVF-FF also having lower levels than IVF(+ve).

**LIMITATIONS, REASONS FOR CAUTION:**

This is an *in vitro* study and caution must be taken when extrapolating these results *in vivo*.

**WIDER IMPLICATIONS OF THE FINDINGS:**

This study reveals important details of impaired [Ca^2+^]_i_ signalling in sperm from sub-fertile men that cannot be detected in population studies.

**STUDY FUNDING/COMPETING INTEREST(S):**

This study was funded by a MRC project grant (MR/M012492/1; MR/K013343/1). Additional funding was provided by Chief Scientist Office/NHS research Scotland.

## Introduction

Sperm dysfunction is commonly regarded as the single most common cause of infertility yet there is a paucity of non-ART treatments available (Martins da Silva *et al.*, 2017). A detailed understanding of the working of the normal and dysfunctional cell is necessary to develop a platform for new diagnostic and treatment options (Barratt *et al.*, 2017, 2018). Intracellular Ca^2+^ ([Ca^2+^]_i_) signalling is fundamental in regulation of many aspects of sperm function including motility and the acrosome reaction (Publicover *et al.*, 2007) and dysregulation of any aspect of sperm [Ca^2+^]_i_ signalling is thought to impair the normal function of sperm and reduce fertilization capability (Krausz *et al.*, 1995; Williams *et al.*, 2015). CatSper, the primary Ca^2+^-influx channel of sperm, is weakly voltage-sensitive and is activated by intracellular alkalinization, but in human sperm is also sensitive to a variety of ligands, the best-characterized of which is progesterone (P4) (Lishko *et al.*, 2011; Strünker *et al.*, 2011). P4 may therefore cause strong activation of the channel as sperm approach the oocyte, the consequent Ca^2+^ influx regulating activities required for fertilization (Lishko *et al.*, 2012). Mouse sperm null for CatSper are sterile (Ren *et al.*, 2001) and previous studies on sperm from ART patients revealed impaired [Ca^2+^]_i_ handling and reduced ability to respond to P4, particularly in samples that subsequently failed to fertilize at IVF, indicating that CatSper lesions may underlie reduced fertility in these men (Krausz *et al.*, 1995, 1996; Alasmari *et al.*, 2013a) Recently Williams *et al.* (2015) combined screening of P4-induced [Ca^2+^]_i_ signals with direct assessment of CatSper currents to show that a complete lack of functional CatSper (no [Ca^2+^]_i_ response to P4 or membrane current) is enough to compromise fertilizing ability and IVF outcome. Interestingly, though only one patient had no detectable CatSper function, several patients had more subtle abnormalities of the [Ca^2+^]_i_ response when challenged with P4 (Williams *et al.*, 2015).

P4 [Ca^2+^]_i_ responses of individual sperm vary greatly within a single ejaculate (Kirkman-Brown *et al.*, 2000). For instance, within a sample the response to P4 of an individual cell may be negligible or may exceed modal amplitude by >2-fold (Kirkman-Brown *et al.*, 2000; Lefièvre *et al.*, 2012). However, all previous studies on CatSper-mediated [Ca^2+^]_i_ responses of ART patients have used fluorimetric techniques that record only the summed response of a large population (Krausz *et al.*, 1995, 1996; Williams *et al.*, 2015). Though showing clearly that [Ca^2+^]_i_ signalling in sub-fertile men is abnormal, this approach provides no information on the distribution of single cell responses in these samples and how this varies compared to that of ‘normal’ (donor) cells.

Although time consuming and technically more complex, single cell [Ca^2+^]_i_ imaging provides information on activity of individual sperm that cannot be obtained by studying populations, including the proportion of responsive cells, the presence of sub-populations that respond differently and the nature and complexity of the single cell [Ca^2+^]_i_ signal. We have used single cell imaging to investigate responses to P4 in sperm samples from sub-fertile men attending an ART clinic, specifically (i) the nature and heterogeneity of single cell [Ca^2+^]_i_ responses and (ii) the relationship between P4-induced [Ca^2+^]_i_ responses and fertilization success.

## Materials and Methods

### Experimental design

Single cell [Ca^2+^]_i_ imaging of spermatozoa from patients was carried out using an aliquot of the sperm preparation used for ART. Measurements were made on the day of treatment, allowing direct correlation with ART. Computer-assisted sperm analysis (CASA) was done on each aliquot. For analysis, patient samples were divided into three groups IVF(+ve) (successful fertilization), IVF-FF (failed fertilization) and ICSI.

### Ethical approval

Written consent was obtained from each patient in accordance with the Human Fertilization and Embryology Authority (HFEA) Code of Practice (version 8) under local ethical approval (13/ES/0091) from the Tayside Committee of Medical Research Ethics B. Similarly, volunteer sperm donors were recruited in accordance with the HFEA Code of Practice (version 8) under the same ethical approval.

### Selection and preparation of spermatozoa

Patients were selected for treatment according to clinical criteria and semen quality: i.e. those with normal sperm concentration and motility (WHO, 2010) and 1 × 10^6^ progressively motile cells post-preparation were selected for IVF, those who failed to meet these criteria were treated by ICSI. 441 patients attended the clinic and provided samples during the study period (January 2016–June 2017) of which 101 were tested. Supplementary Information Fig. S7 presents the flowchart of patients and reasons for inclusion/exclusion. The surplus clinical sample used in the IVF/ICSI treatment was used where consent was given. Control semen samples were obtained from volunteer donors with normal sperm concentration, motility and semen characteristics (WHO, 2010) and no known fertility problems. Samples were obtained by masturbation after 48–72 h of sexual abstinence.

Patient cells were prepared according to the standard operating procedures employed by the ACU and donor cells were prepared in an identical fashion but with equivalent bicarbonate buffered sperm capacitation medium prepared in house (Brown *et al.*, 2016). After 30 min of liquefaction at 37°C, donor and patient sperm were isolated using a discontinuous density gradient procedure (Tardif *et al.*, 2014; Williams *et al.*, 2015). Up to 2 ml of semen was loaded on top of a 40–80% suspension of Percoll (Sigma Aldrich, UK;) diluted with HEPES buffered saline (donor semen) or Pureception (colloidal silica suspension for sperm preparation; Origio, Denmark) diluted with Spermwash (Origio, Denmark; patient semen). The density gradient was then centrifuged at 300 *g* for 20 min, washed (300 *g*, 10 min) and re-suspended in bicarbonate buffered sperm capacitation medium or Quinn’s advantage human tubal fluid (HTF) (Origio, Denmark) (donor and patients, respectively). All samples were left to capacitate at 37°C, 95% O_2_/5% CO_2_ for a 5–7 h prior to experimentation. Samples were obtained and analysed in line with suggested guidance for human semen studies where appropriate (Björndahl *et al.*, 2016). To assess whether [Ca^2+^]_i_ responses were affected by preparation protocol, control experiments on donor cells were carried out in which semen samples were split and prepared in parallel as described above using IVF clinic medium for one aliquot and bicarbonate buffered sperm capacitation medium for the other. P4-induced Ca^2+^ signals were similar in cells prepared by the two methods (Supplementary Information Fig. S1).

### Single cell [Ca^2+^]_i_ imaging

Sperm were prepared and assessed as previously described (Brown *et al.*, 2017). Briefly, capacitated sperm (1–2 million cells/ml) were loaded with 2 μM Fluo-4 (Molecular Probes, UK) at 37°C for 20 min then centrifuged at 300 *g* for 10 min. The supernatant was removed and pellet re-suspended in supplemented Earle’s balanced salt solution (sEBSS). This wash step was repeated and the pellet was re-suspended in sEBSS for imaging. Sperm were loaded into a small-volume imaging chamber (RC-20, Harvard apparatus UK) sealed with vacuum grease (DowCorning 976) on a poly-d-lysine (0.05%) coated coverslip, and allowed to adhere for ~5 min. Experiments were performed at 33 ± 0.5°C in a continuous flow of sEBSS solution. A 10 min wash period was allowed before imaging commenced. After recording resting [Ca^2+^]_i_ levels for 3–5 min, cells were stimulated with P4 (3.6 μM). Images were acquired at 0.33 Hz using a ×40 oil objective with a CoolSNAP MYO CCD camera controlled by Metsoftware (Molecular Devices, USA). Fluorescence was excited at 488 nm and recorded at 520 nm. Illumination and camera gain settings were maintained constant and fluorescence intensity values are therefore directly comparable between all recordings. A region of interest was drawn round the head and neck region of each cell and several areas were also chosen to assess background fluorescence. Those cells where fluorescence levels fell noticeably during the pre-stimulation period (loss of dye indicating that the cell was dead or dying) were excluded from the analysis. After background correction, resting fluorescence intensity (mean of 25–30 consecutive images collected prior to P4 stimulation) and peak fluorescence intensity (mean of 4–5 consecutive images spanning the peak of the P4-induced [Ca^2+^]_i_ transient) were determined for each cell. P4-induced fluorescence increment for each cell was then calculated by subtracting resting fluorescence from peak fluorescence (Supplementary Information Fig. S2). Normalization of background-corrected fluorescence data was as described previously (Alasmari *et al.*, 2013a,b) using Δ*F* = ((*F* − *F*_rest_)/*F*_rest_) × 100%, where Δ*F* is percentage change in intensity, *F* is fluorescence intensity at time *t*, and *F*rest is the mean of 25–30 determinations of *F* prior to P4-stimulation. A mean normalized trace was calculated for each experiment by taking the mean Δ*F* of all cells in the experiment (Δ*F*_mean_) at each time point. To assess responsiveness to P4 in each cell, the mean and 95% confidence interval of fluorescence intensity were calculated for the period prior to P4 stimulation (C ± c) and the 4–5 images spanning the peak of the transient response (*T* ± *t*). The response of that cell was considered significant and classified as a responder if: *T* – *t* > *C *+ *c* (Kirkman-Brown *et al.*, 2000).

### Single cell [Ca^2+^]_i_ oscillations

To assess the occurrence of [Ca^2+^]_i_ oscillations in patients and donors, traces were examined by eye for the occurrence of cyclical increases in [Ca^2+^]_i_. In 10 patient samples, spontaneous [Ca^2+^]_i_ oscillations were observed during the control period (prior to P4 application) which persisted in the presence of P4. These oscillations often ‘masked’ the [Ca^2+^]_i_ response to P4 which could not be assessed. These data are presented and discussed separately and are not included in the 3 patient groups.

### Fertilization rate at IVF

Oocytes were considered normally fertilized when two pronuclei formed (2PN) and two polar bodies were observed. In IVF, the fertilization rate was calculated from the number of oocytes normally fertilized divided by the total number of inseminated oocytes. Fertilization rate for IVF was calculated only when four or more mature oocytes (metaphase II) were present.

### Failed fertilization

Patients were classified as failed fertilization (IVF-FF) when no pronuclei were observed after insemination (minimum of four eggs for inclusion of study). Experimentation (CASA, single cell imaging) was carried out on the day of insemination and therefore the status of the outcome of IVF treatment was unknown. No ICSI FF patients were included in IVF-FF analysis.

### Sperm kinematics

A Hamilton Thorne CEROS computer aided sperm analysis machine was used to measure sperm sample kinematics and hyperactivation of prepared samples from ART patients (where sufficient sample was available) and donors (Alasmari *et al.*, 2013a).

### Experimental solutions

Composition of experimental solutions: HEPES buffered saline, bicarbonate buffered capacitating medium and sEBBSS are as follows:

HEPES buffered saline solution consisted of (in mM): CaCl_2_, 1.8; KCl, 5.4; MgSO_4_7H_2_O, 0.8; NaCl, 116.4; NaH_2_PO_4_, 1; d-glucose, 5.5; sodium pyruvate, 2.73; sodium lactate, 41.75; HEPES, 25; BSA, 0.3% (w/v); pH adjusted to 7.4 using NaOH. Bicarbonate buffered capacitating medium consisted of (in mM): CaCl_2_, 1.8; KCl, 5.4; MgSO_4_7H_2_O, 0.8; NaCl, 116.4; NaH_2_PO_4_ 1; d-glucose, 5.5; sodium pyruvate, 2.73; sodium lactate, 41.75; sodium bicarbonate, 26; BSA, 0.3% (w/v); pH adjusted to 7.4 using NaOH.
Supplemented Earle’s balanced salt solution (sEBSS) contained (in mM); NaH_2_PO_4_, 1.02; KCl, 5.4; MgSO_4_, 0.811; d-glucose, 5.5; Na pyruvate, 2.5; Na lactate, 19.0; CaCl_2_,1.8; NaHCO_3_, 25.0; NaCl, 118.4 and HEPES, 15 (pH 7.4), supplemented with 0.3% (w/v) BSA.

### Data analysis

For analysis, patient samples were divided into three groups IVF(+ve) (successful fertilization; 62 samples), IVF-FF (failed fertilization; eight samples) and ICSI (21 samples, which included three samples from patients who had previously failed to fertilize at IVF). Data were analysed using Microsoft Excel™ or GraphPad Prism™ (version 5, GraphPad Software Inc.). Data were assessed for normality using the Shapiro–Wilk test. Statistical significance was determined using Student’s *t*-test, Chi-square, Kruskal–Wallis test or ANOVA as appropriate. Regression analyses of fluorescence increment:resting fluorescence were carried out in Excel using the ‘set intercept = zero’ option. Regression coefficients were compared as described by Clogg *et al.* (1995) and corrected *post hoc* for multiple comparisons (Gaetano, 2013). Percentage data were converted using the arcsine square root transformation (Sokal and Rohlf, 1981) before statistical analysis to allow application of parametric tests. Holm–Bonferroni correction (Gaetano, 2013) *post hoc* correction was applied as appropriate. Data are presented as mean ± SEM with *P* < 0.05 indicative of statistical significance.

## Results

### Resting [Ca^2+^]_i_ in donor and patient cells

**Figure 1:**
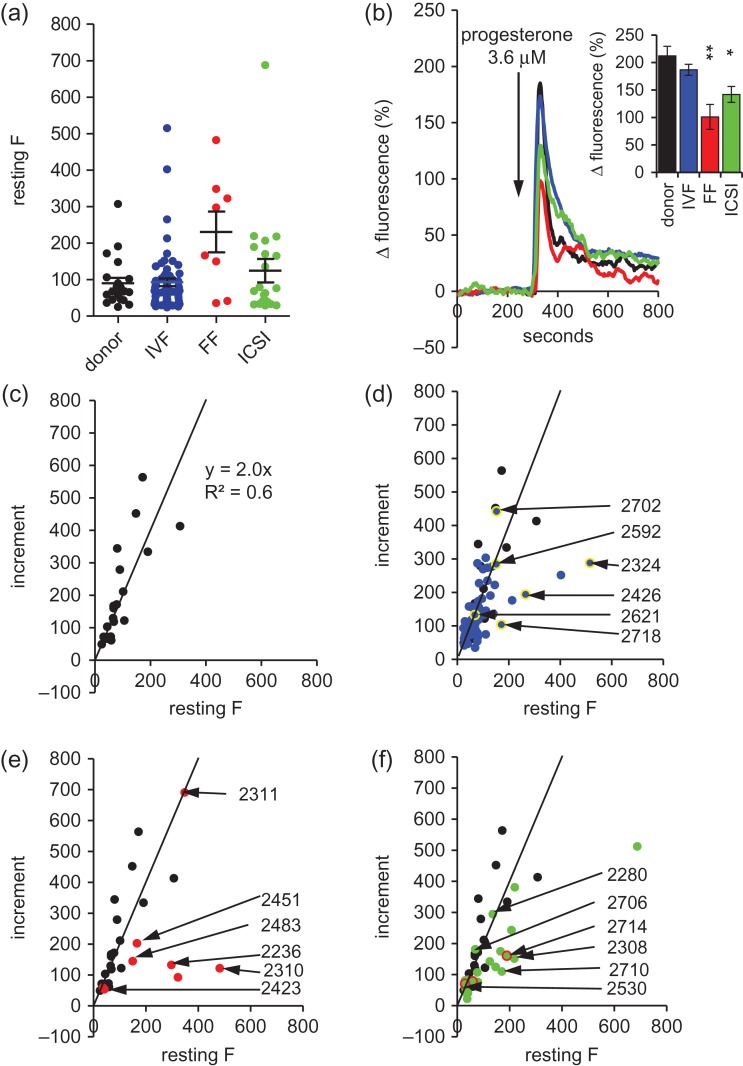
Resting fluorescence and population responses to P4. (**a**) Mean resting fluorescence for donor (black; *n* = 21 samples), IVF(+ve) (blue; *n* = 62 samples), IVF-FF (red; *n* = 8 samples) and ICSI (green; *n* = 21 samples) groups. Plots show individual values and mean ± SEM. (**b**) [Ca^2+^]_i_ responses to P4 in donors (black), IVF(+ve) (blue), IVF-FF (red) and ICSI (green) groups. Arrow shows time of progesterone addition. Plots were obtained by normalizing data for each cell to pre-stimulus level, calculating the population response (mean of all cells imaged − Δ*F*_mean_) for each sample and then averaging these for the donors (*n* = 21 experiments) and for each of the three patient groups: IVF(+ve) (*n* = 62 experiments), IVF-FF (*n* = 8 experiments) and ICSI (*n* = 21 experiments). Inset shows mean (± SEM) normalized transient amplitude for each data set. Asterisks indicate *P* < 0.05 (*) and *P* < 0.01 (**) with respect to donor samples. (**c**) Relationship between mean resting fluorescence and mean fluorescence increment for 21 donor samples. Line shows fitted regression (*y* = 2.0*x*; *R*^2^ = 0.6). (**d**–**f**) Relationship between mean resting fluorescence and mean fluorescence increment for IVF(+ve) ((d) blue, *n* = 62 samples); IVF-FF ((e) red; *n* = 8 samples) and ICSI ((f) green, *n* = 21 samples), respectively. Numbered points (highlighted yellow in panel (d) for clarity) show patients for whom single cell analysis is shown in Fig. 2 and Supplementary Information Figs S3–S5. Points highlighted in red in panel (f) (ICSI) are patients who had previously failed to fertilize any oocytes at IVF. In each of panels (d–f) black points and fitted regression show data from donor samples for comparison.

Mean resting [Ca^2+^]_i_ levels (fluo4 fluorescence after background correction) were similar in donors, IVF+ (successful fertilization) and ICSI patients, but in the eight IVF-FF (failed fertilization) patients mean resting fluorescence was more than double that in donor cells (Fig. 1a). Examination of variation within the four categories showed that the majority of donor samples clustered in the range 25–200 and just 1/21 samples (4.8%) exceeded 250. In IVF(+ve) and ICSI populations the proportion of samples with a mean resting fluorescence >250 was similar (4.8%) but 50% (4/8) of IVF-FF samples exceeded this value (*P* = 0.004; *P* = 0.002 and *P* = 0.004 compared to donor, IVF(+ve) and ICSI samples, respectively; Chi-square; Fig. 1a).

### [Ca^2+^]_i_ responses to P4

As described previously (Kirkman-Brown *et al.*, 2000; Williams *et al.*, 2015), stimulation of human sperm with 3.6 μM P4 induced a biphasic [Ca^2+^]_i_ signal composed of an initial transient followed by a sustained [Ca^2+^]_i_ plateau (Fig. 1b). Initially we analysed the data by normalizing fluorescence of fluo4 to the pre-stimulus (resting) level and calculating a mean normalized response for each experiment (Δ*F*_mean_; see Materials and Methods). Using this approach the amplitudes of [Ca^2+^]_i_ transients in samples from ICSI patients and IVF-FF patients were significantly lower than those of donors (Fig. 1b, inset). However, since high levels of resting fluorescence were observed in a large proportion of IVF-FF samples (see above), this approach is potentially misleading since, at high resting [Ca^2+^]_i_, an equivalent P4-induced [Ca^2+^]_i_ increment will result in a smaller normalized response and also [Ca^2+^]_i_ may approach levels at which dye saturation occurs. To investigate this we examined the relationship between resting fluorescence and the P4-induced fluorescence increment. Plotting of mean transient amplitude (increment in fluorescence intensity) against mean resting [Ca^2+^]_i_ (resting fluorescence) for each of the 21 donor recordings gave an approximately linear relationship (*y* = 2.00*x*; *R*^2^ = 0.6; Fig. 1c) over a range of resting fluorescence from 25 to >300. Plotting of equivalent data for the 62 IVF(+ve) samples gave a more complex plot. Most points fell on a straight line very similar to that for donor samples (Fig. 1d), but in a number of samples (≈10%) the mean fluorescence increment fell below the ‘expected’ range (Fig. 1d). Similar analysis of the IVF-FF and ICSI patients also showed variation between samples in responsiveness to P4 (Fig. 1e and f). Overlaying these plots with the data for donor experiments clearly showed that, for a given mean resting fluorescence the mean P4-induced [Ca^2+^]_i_ transient in some ICSI samples and most IVF-FF samples was smaller (Fig. 1e and f).

**Figure 2:**
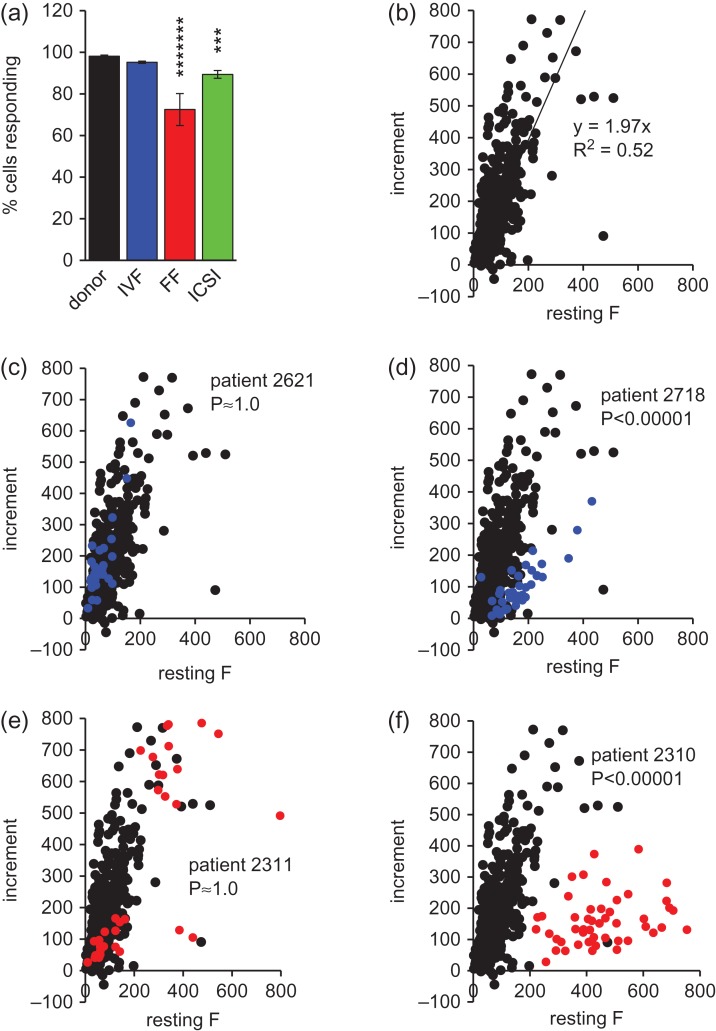
Single cell P4-induced [Ca^2+^]_i_ transients. (**a**) Proportion of cells showing significant increase in fluorescence upon application of 3.6 μM P4. Asterisks indicate *P* < 0.005 (***) and *P* < 0.00005 (*******) with respect to donor samples. (**b**) Relationship between resting fluorescence and fluorescence increment for 749 cells from 21 donor samples. Line shows fitted regression (*y* = 1.97*x*; *R*^2^ = 0.52). (**c**–**f**) Show examples of relationship between mean resting fluorescence and mean fluorescence increment in two IVF(+ve) patients ((c) and (d); 32 and 37 cells, respectively; blue symbols); and two IVF-FF patient ((e) and (f) 52 and 53 cells, respectively; red symbols). In each of panels (c–f) black points and show data from donor cells (b) for comparison. Numbers in each panel are patient code (for comparison with Fig. 1) and *P* values show comparison of patient regression coefficient with that for donor cells.

To assess the variation of single cell responses to P4, [Ca^2+^]_i_ transient amplitude was assessed in each cell. In donor samples almost all cells (98.1 ± 0.5%) generated a significant increase in fluorescence upon stimulation with P4 (Fig. 2a). The great majority of cells in patient samples were also responsive but the proportion was significantly lower in all three groups, particularly in the IVF-FF (72.5 ± 7.7%; *P* < 0.00005; Fig. 2a). Plotting of transient amplitude (increment in fluorescence intensity) against resting [Ca^2+^]_i_ (resting fluorescence) for each of the 749 donor cells (21 samples) gave a straight line relationship with a gradient of ~2 (y = 1.97x; *R*^2^ = 0.52), similarly to that obtained when plotting of mean data for each experiment (compare Fig. 1c and Fig. 2b). Overlay of single cell data from IVF(+ve) patient samples showed that whereas most samples followed the distribution seen with donor cells (e.g. Fig. 2c; Supplementary Information Fig. S3a and b), in samples where the mean response deviated from the distribution of donor samples (Fig. 1d) single cell responses clearly diverged from the distribution of donor cells, even when resting fluorescence was well within the ‘normal’ range (Fig. 2d; Supplementary Information Fig. S3c and d). Fitting of linear regressions to single cell distributions confirmed that that these differences were significant (Fig. 2, Supplementary Information Fig. S3). Single cell analysis and fitting of linear regressions to single cell distributions of ICSI and (more particularly) IVF-FF cells showed considerable variability between samples, consistent with the scatter of mean values shown in Fig. 1e and f. In the samples from IVF-FF patients 2310 and 2236, most cells, including those with the lowest resting fluorescence, deviated strongly from the donor distribution resulting in a significantly different regression coefficient (Fig. 2f, Supplementary Information Fig. S4b; *P* < 0.00001). In contrast, several of the other IVF-FF samples had distributions much closer to that for donor cells (Supplementary Information Fig. S4a, c and d) and the distribution for patient 2311 was indistinguishable (*P* ≈ 1.0 compared to donor cells; Fig. 2e). Single cell distributions for ICSI samples showed similar variability (Supplementary Information Fig. S5). The 21 ICSI samples included three that were from men who had previously failed to fertilize any oocytes at IVF (highlighted red in Fig. 1f). In one of these patients (2714) [Ca^2+^]_i_ responses to P4 deviated markedly from the distribution for donor cells (Supplementary Information Fig. S5e), but the other two samples (2508, 2530) fell close to the donor distribution (Fig. 1f, Supplementary Information Fig. S5f). Overall, examination of single cell plots of poorly responsive samples from all three patient groups indicated that the small P4-induced increment was a genuine characteristic of the population and was not specifically associated with high levels of resting fluorescence (high resting [Ca^2+^]_i_).

**Figure 3:**
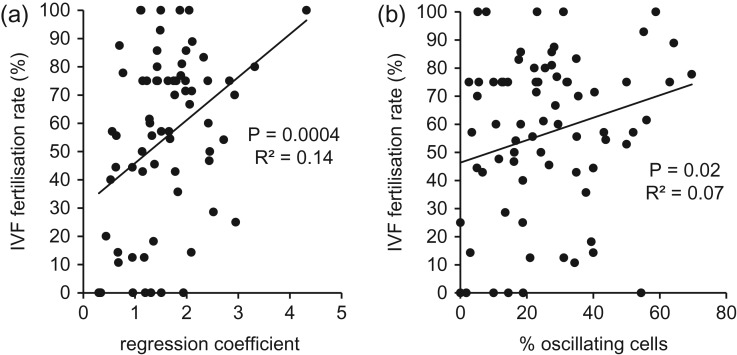
[Ca^2+^]_i_ signalling and fertilization rate. (**a**) P4 sensitivity and IVF fertilization rate. *X*-axis is regression coefficient calculated for the relationship between single cell resting fluorescence and P4-induced fluorescence for each sample. *Y*-axis vale is fertilization rate for each sample. Data from 62 IVF(+ve) and 8 IVF-FF samples. (**b**) Induction of [Ca^2+^]_i_ oscillations by P4 and IVF fertilization rate. Plot shows relationship between proportion of cells in which oscillations were induced by treatment with P4 (3.6 μM) and the fertilization rate (%) achieved at IVF with that sample. Data from 62 IVF(+ve) and 8 IVF-FF samples.

Since IVF patient samples varied considerably in their sensitivity to P4, we investigated the relationship between the regression coefficient (P4-induced fluorescence increment:resting fluorescence) for each sample and fertilization rate of that sample at IVF. There was a significant positive relationship between these variables (*P* = 0.0004; *R*^2^ = 0.14; Fig. 3a). Furthermore, separation of IVF samples into those with a regression coefficient <1.0 (increment in fluorescence less than resting fluorescence) and those with a coefficient of ≥1.0 gave mean fertilization rates of 31.0 ± 7.6% (*n* = 55) and 61.8 ± 3.8% (*n* = 15), respectively (*P* = 0.0015).

#### Occurrence of P4-induced [Ca^2+^]_i_ oscillations

**Figure 4:**
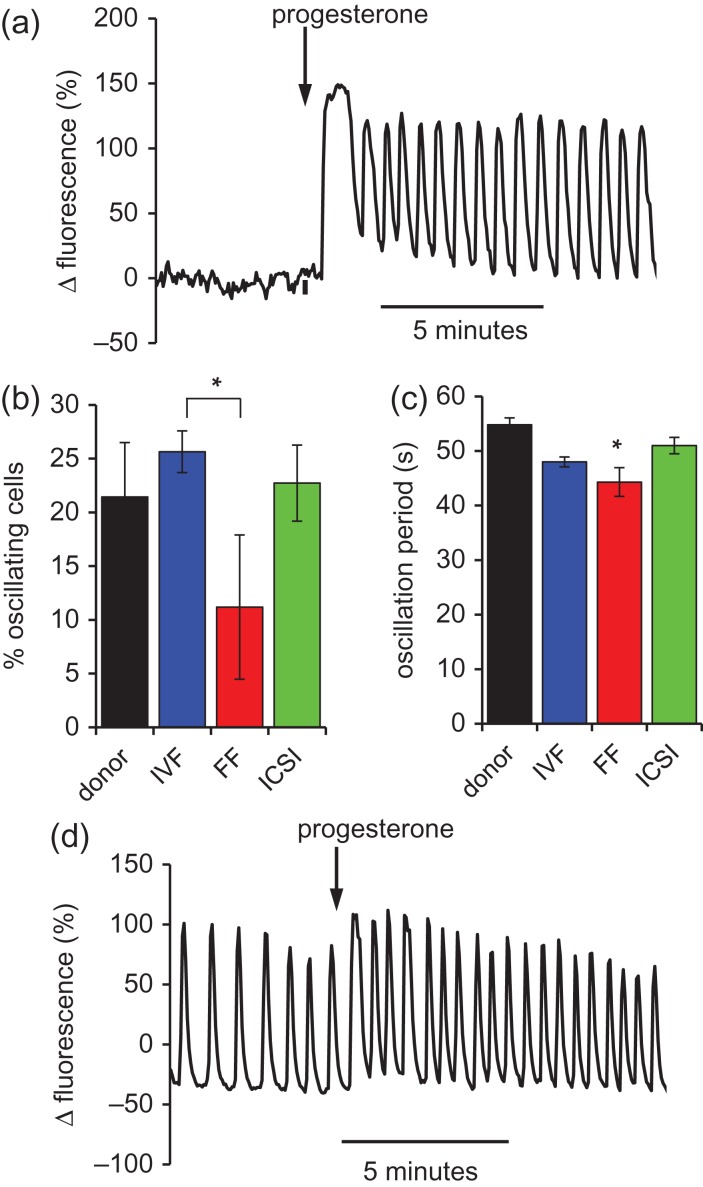
Calcium oscillations in progesterone-stimulated cells. (**a**) Representative trace of a P4-induced [Ca^2+^]_i_ oscillation in single spermatozoon of an IVF (+ve) patient. 3.6 μM P4 was added at the arrow. (**b**) Proportion of cells that generated [Ca^2+^]_i_ oscillations when stimulated with 3.6 μM P4. Bars show mean ± SEM for donors (black, *n* = 21 experiments), IVF(+ve) (blue, *n* = 62 experiments), and IVF-FF (red, *n* = 8 experiments and ICSI (green, *n* = 21 experiments). *P* < 0.05 (*). (**c**) Mean [Ca^2+^]_i_ oscillation period (± SEM); donors (black, *n* = 143 cells), IVF(+ve) (blue, *n* = 582 cells), IVF-FF (red, *n* = 43 cells; *P* < 0.05 with respect to donors (*)) and ICSI (green, *n* = 162 cells). (**d**) Example of cell (successful IVF patient) generating large spontaneous [Ca^2+^]_i_ oscillations, which persisted during P4 exposure.

Single cell imaging allows the detection of complex [Ca^2+^]_i_ signals that are masked in populations measurements. A common observation is the occurrence of [Ca^2+^]_i_ oscillations, superimposed on the plateau phase of the P4-induced [Ca^2+^]_i_ response (Fig. 4a). Figure 4b shows the proportion of cells in which P4 induced [Ca^2+^]_i_ oscillations occurred. In all three patient groups we observed induction of [Ca^2+^]_i_ oscillations upon stimulation with P4 but whereas frequency of occurrence in IVF(+ve) and ICSI samples was 20–25%, similar to donor controls (21.4 ± 5.0%, *n* = 22; Fig. 4b), in IVF-FF samples the proportion of oscillating cells was only 11.2 ± 6.7% (*n* = 8). Variation between the eight IVF-FF patients was considerable (proportion of oscillating cells ranged from 0 to 54%), but the proportion of samples in which no cells generated [Ca^2+^]_i_ oscillations (3/8) significantly exceeded that in donors (1/21; *P* < 0.02) or IVF(+ve) samples (2/62; *P* < 0.0005). Plotting of the relationship between generation of [Ca^2+^]_i_ oscillations (% cells oscillating) and fertilization for all IVF samples (IVF(+ve) and IVF-FF) revealed a weak but significant correlation (*P* = 0.02; *R*^2^ = 0.054; Fig. 3b). In all patient groups the period of P4-induced oscillations was slightly shorter than in controls, but this difference was significant only in the IVF-FF group, where oscillation period was 44.3 ± 2.6 s (*n* = 48 cells) compared to 54.8 ± 1.3 s (*n* = 183 cells) in donors (Fig. 4c; *P* < 0.05).

#### Spontaneous calcium oscillations

In ≈ 8% of donor cells (63/749) we observed spontaneous [Ca^2+^]_i_ oscillations, as described previously (Sanchez-Cardenas *et al.*, 2014) but amplitudes were small compared to those induced by P4 (Δfluorescence = 31 ± 3.5% and 113 ± 26%, respectively; *P* < 0.001). However, in 10 patient samples, all of which fertilized at IVF (fertilization rate = 60.7 ± 7.4%), we observed large spontaneous [Ca^2+^]_i_ oscillations similar in amplitude to those induced by P4 (Fig. 4d). These patients were not included in analysis of P4-induced [Ca^2+^]_i_ signalling because spontaneous activity masked/distorted the response to P4 (Fig. 4d). Stimulation with P4 caused an increase in baseline [Ca^2+^]_i_ but spontaneous oscillations persisted and no clear P4-induced transient could be discerned (Fig. 4d). Neither the amplitude nor the frequency of these spontaneous [Ca^2+^]_i_ oscillations was significantly altered in the presence of P4 (*P* > 0.05). Examination of the relationship between the proportion of spontaneously oscillating cells in each of these 10 patients and fertilization rate at IVF showed a weak, non-significant relationship (*P* = 0.19; Supplementary Information Fig. S6).

### Motility of patient and donor sperm

**Figure 5:**
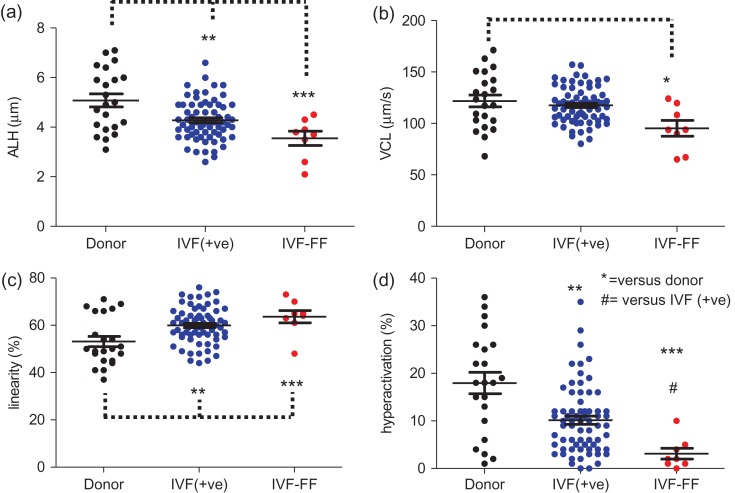
Kinematics and hyperactivation of donor and patient cells (assessed by CASA). Plots show mean ± SEM and distribution of individual values for: (**a**) amplitude of lateral head movement (ALH; μm); (**b**) curvilinear velocity (VCL; μm/s); (**c**) linearity (%); and (**d**) hyperactivation (%). Asterisks indicate statistical difference from donors except where indicated *P < 0.05. **P < 0.01, ***P < 0.001.

All donor and IVF patient samples included in this study were assessed by CASA prior to experimentation. Due to the volume and cell concentration of most ICSI samples, accurate CASA analysis was not possible. Analysis of motility data (total and progressive) showed no significant differences between donor and patient populations, but motility kinematics were clearly altered in patient samples. Figure 5 shows the distributions of amplitude of lateral head movement (ALH) (panel a), curvilinear velocity (VCL) (panel b), linearity (panel c) and percentage of hyperactivated cells (panel d) for the donor, IVF(+ve) and IVF-FF groups. Patient samples had higher linearity and lower ALH and VCL (IVF-FF only). Consistent with these differences, both IVF(+ve) (10.2 ± 0.9%, *n* = 62) and IVF-FF (3.1 ± 1.1%, *n* = 8) had a significantly lower percentage of hyperactivated cells when compared to donor samples (18.0 ± 2.3%, *n* = 21; *P* = 0.00005 and 0.0007, respectively). The percentage of hyperactivated cells in IVF-FF samples was also significantly lower than in the IVF(+ve) group, *P* = 0.02.

## Discussion

CatSper channels are the main source of Ca^2+^ entry in human sperm (Brenker *et al.*, 2012), and studies in which CatSper activity and fertility (outcome of IVF treatment) of sperm populations have been assessed suggest that even minor abnormalities of CatSper function may affect fertility (Krausz *et al.*, 1995, 1996; Qi *et al.*, 2007; Lishko and Kirichok, 2010; Williams *et al.*, 2015). However, assessment of CatSper function in sperm populations masks the occurrence of cell–cell variation within the sample which may be of functional or diagnostic significance. We used single cell imaging to explore the heterogeneity of single cell [Ca^2+^]_i_ responses to P4 in donor and patient samples and to assess how this relates to fertilizing ability (by IVF) of the sperm population. Our data show not only that P4-evoked and spontaneous [Ca^2+^]_i_ signals vary between cells in a single ejaculate (as has been described previously for cells from ‘healthy’ donors), but that there is clear variation between and within patient types (as assessed by an ART clinic) in regard to the proportion of cells that respond to the CatSper agonist P4 and the nature of the responses elicited.

### Resting and P4-stimulated [Ca^2+^]_i_ in donor and patient sperm

Analysis of resting (pre-stimulus) fluorescence showed wide variation between samples both within and between patient and donor groups. In particular, in the IVF-FF patient group, half of the samples showed an unusually high resting fluorescence. Though we cannot discount the possibility that this reflects abnormalities of dye loading/behaviour in these samples, it suggests that high resting sperm [Ca^2+^]_i_ may be characteristic of some sub-fertile men. Increased resting [Ca^2+^]_i_ could be due to enhanced tonic Ca^2+^-influx through CatSper, for instance due to unusually high pH_i_ or depolarized Vm (Brown *et al.*, 2016). Alternatively, impairment of Ca^2+^ clearance mechanisms may cause elevated resting [Ca^2+^]_i._ For instance, sperm from plasma membrane calcium ATPase 4 (PMCA4)-null mice have increased [Ca^2+^]_i_, though the loss of motility in such cells is far more severe than the effects observed in this study (Okunade *et al.*, 2004; Schuh *et al.*, 2004).

Since resting fluorescence varied between donor/patient groups, simple normalization of fluorescence to pre-stimulus levels is potentially misleading. If the high levels of resting fluorescence in these samples genuinely reflect high [Ca^2+^]_i_ then (i) a ‘normal’ P4-induced CatSper activation/Ca^2+^ influx will give a smaller proportional increase in fluorescence and (ii) the dye may approach saturation, underestimating the [Ca^2+^]_i_ signal. Therefore, to analyse the amplitude of [Ca^2+^]_i_ responses to progesterone we investigated the relationship between resting fluorescence and the P4-induced fluorescence increment. Plotting the data from donor samples either using sample means or individual cells gave a clear, linear relationship that showed no evidence of dye saturation over the range of resting fluorescence observed. For most patient samples a similar relationship between resting fluorescence and the P4-induced fluorescence increment was seen but in ~10% of IVF(+ve) patients and one-third of IVF-FF and ICSI patients the response to P4 fell clearly below the ‘normal’ range.

Examination of the single cell resting fluorescence: P4-induced increment plots from samples which gave ‘sub-normal’ responses to P4 suggests that the nature of the underlying lesion varies. In each of the patient groups we observed some samples that generated clearly linear scattergrams but responses to P4 were smaller than those obtained with donor sperm, such that the gradient of the plot was significantly lower. Such reduced sensitivity could occur due to poor expression of CatSper channels (Tamburrino *et al.*, 2015). Alternatively, the expression of a mutant CatSper channel with reduced conductance, as has recently been described for mouse sperm lacking CatSperζ (Chung *et al.*, 2017), could produce this phenotype. A second pattern seen in patient’s samples was a ‘cloud’ of points to the right of/below the donor distribution. Resting fluorescence was unusually high in some of these samples, but it is also notable that the ratio of P4-induced increment to resting fluorescence varied greatly between cells, indicating great intra-sample variation in resting [Ca^2+^]_i_ and/or expression of functional CatSper. Data from patient 2236 produced an intriguing ‘hybrid’ plot including cells that responded ‘normally’ to P4 and cells that gave a negligible/zero response, suggesting that only a sub-population of these sperm express functional CatSper. Significantly, though the response to P4 was impaired in a significant proportion of the 91 patients where analysis was possible, we did not detect any men who were null or ‘functionally null’ (Williams *et al.*, 2015) for CatSper in every cell, indicating that such patients are very rare.

### P4-induced [Ca^2+^]_i_ signalling and fertility

To assess the functional significance of this variability in response to P4, we examined the relationship between P4-sensitivity (regression coefficient of the single cell scatter plot) and fertilization rate of the sample in IVF. Consistent with previous studies on P4-induced population [Ca^2+^]_i_ signals (Krausz *et al.*, 1995, 1996; Alasmari, *et al.*, 2013a; Williams *et al.*, 2015), the data showed a significant positive relationship. Taken together with our observation that most IVF patients had a high proportion of cells in which a significant response to P4 was detected (mean ≈ 95%), this suggests the existence of a threshold level of single sperm CatSper activity/P4 sensitivity below which fertilization competence of the cell is compromised. Notably, some IVF-FF samples responded ‘normally’ or near-normally to P4—failure of such samples to fertilize probably reflects lesions not associated with [Ca^2+^]_i_ signalling.

### [Ca^2+^]_i_ oscillations in donors and patient sperm

Upon stimulation of human sperm with P4, the initial [Ca^2+^]_i_ transient is followed, in a subset of cells, by [Ca^2+^]_i_ oscillations which are dependent on influx of extracellular Ca^2+^ but appear also to involve repetitive mobilization of Ca^2+^ stores (Harper *et al.*, 2004; Kirkman-Brown *et al.*, 2004; Bedu-Addo *et al.*, 2007; Sanchez-Cardenas *et al.*, 2014; Mata-Martínez *et al.*, 2018). These oscillations are reported both to regulate activity of the flagellum, potentially modifying sperm behaviour to facilitate penetration of the oocyte vestments (Harper *et al.*, 2004), and to be associated with low levels of acrosome reaction (Harper *et al.*, 2004; Sanchez-Cardenas *et al.*, 2014). In this study P4-induced [Ca^2+^]_i_ oscillations were observed in cells of donors and all patient groups. However, in the failed fertilization (IVF-FF) group the mean percentage of cells that generated oscillations upon P4 treatment was only half that in donors and in the IVF(+ve) group and the proportion of samples that failed totally to generate oscillations was significantly higher in the IVF-FF group. [Ca^2+^]_i_ responses to P4 were small in these samples, consistent with the dependence of oscillations on background Ca^2+^ influx through CatSper. However, in the large IVF(+ve) group (*n* = 62) generation of oscillations showed no significant relationship to P4-induced fluorescence increment (*P* = 0.55; *R*^2^ = 0.006) or to the regression coefficient of the single cell (fluorescence increment:resting fluorescence) scatter plot (*P* = 0.09; *R*^2^ = 0.05), suggesting that other aspects of Ca^2+^-handling, presumably including activity of the Ca^2+^-store, are also important and may lead to failure of oscillations and reduced fertility.

Samples from 10 IVF patients included sperm that showed large spontaneous [Ca^2+^]_i_ oscillations that persisted in the presence of P4 with no significant change in amplitude or frequency and largely masked the P4-induced [Ca^2+^]_i_ transient. The occurrence of spontaneous oscillations might indicate attainment of an advanced level of capacitation (Baldi *et al.*, 1991; Mendoza *et al.* 1993; Garcia and Meizel, 1999; Kirkman-Brown *et al.*, 2000). If this is correct, the variation in their occurrence reflects innate differences between samples since all IVF patient samples were prepared and their responses assessed in the same way. Sanchez-Cardenas *et al.* (2014) reported recently that 98% of cells generating spontaneous [Ca^2+^]_i_ oscillations fail to undergo acrosome reaction upon stimulation with P4, and concluded that this spontaneous activity may suppress premature occurrence of acrosome reaction, though mechanisms are still unknown. All patients in which these large, spontaneous [Ca^2+^]_i_ oscillations were observed successfully fertilized at IVF.

### Impaired [Ca^2+^]_i_ signalling and sperm function

P4-induced (CatSper-mediated) Ca^2+^ influx and P4-induced [Ca^2+^]_i_ oscillation were statistically associated with poor fertilization at IVF. Both these aspects of Ca^2+^ signalling have been implicated in regulation of human sperm motility. Analysis of CASA recordings from the samples used in this study showed significant differences in kinematics between donor cells and the IVF-FF samples. These findings strongly support previous reports of reduced hyperactivation in sub-fertile patients (Alasmari *et al.*, 2013a) and suggest that the relationship between impaired activation of CatSper, abnormal [Ca^2+^]_i_ signalling and poor IVF success rate reported here (and in previous studies on population responses; Krausz *et al.*, 1995, 1996; Alasmari, *et al.*, 2013a) reflects, at least in part, the effect of compromised [Ca^2+^]_i_ signalling on regulation of sperm motility (Alasmari *et al.*, 2013b). However, impaired [Ca^2+^]_i_ signalling is likely also to affect capacitation, regulation of acrosome reaction and viability. We have observed striking differences between patient samples in resting [Ca^2+^]_i_, single-cell P4-sensitivity and generation of [Ca^2+^]_i_ oscillations; future studies should consider the relative incidence, underlying causes and functional significance of these abnormalities for human male fertility.

## Supplementary Material

Supplementary Figure 1

Supplementary Figure 2

Supplementary Figure 3

Supplementary Figure 4

Supplementary Figure 5

Supplementary Figure 6

Supplementary Figure 7
