# Supplementary material for: Single-cell analysis of [Ca2+]i signalling in sub-fertile men: characteristics and relation to fertilization outcome
Source: Hum Reprod. 2018 Apr 25;33(6):1023–33. doi: 10.1093/humrep/dey096 (PMC5972555; doi:10.1093/humrep/dey096)
Supplement: Supplementary Figure 1 [file dey096suppl_figure1.pdf]

Human Reproduction, 2018
doi:10.1093/humrep/dey096

human reproduction

SUPPLEMENTARY DATA

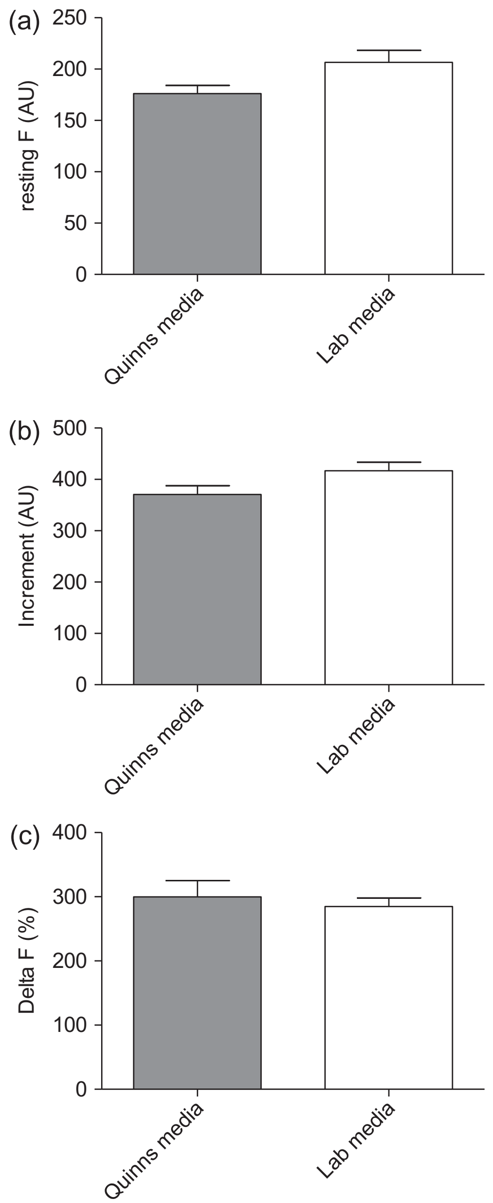

Supplementary Figure S1 Comparison of calcium signalling in donor sperm that were capacitated in commercially available Quinn's media or laboratory prepared bicarbonate-buffered media. Data are from split (paired) samples. Sperm were isolated using a 40–80% Percoll discontinuous density gradient procedure in a HEPES-buffered, BSA-free saline. Mean resting fluorescence (**a**) and P4-induced $[Ca^{2+}]_i$ increase expressed as fluorescence increment (**b**) or normalized to pre-stimulus levels (delta *F* % (**c**)), were not significantly different between treatment groups. Data are from 137 (Quinn's medium) and 157 (laboratory medium) cells from 4 donors.
