# Supplementary material for: Single-cell analysis of [Ca2+]i signalling in sub-fertile men: characteristics and relation to fertilization outcome
Source: Hum Reprod. 2018 Apr 25;33(6):1023–33. doi: 10.1093/humrep/dey096 (PMC5972555; doi:10.1093/humrep/dey096)
Supplement: Supplementary Figure 2 [file dey096suppl_figure2.pdf]

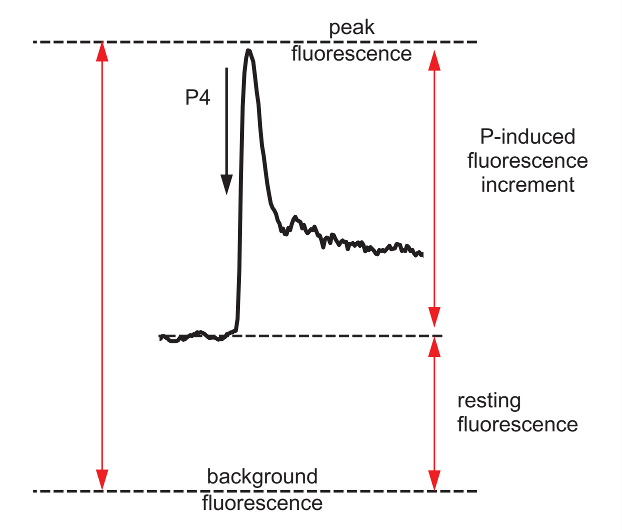

Supplementary Figure S2 Calculation of resting fluorescence and fluorescence increment. After background correction, resting fluorescence intensity (mean of 25–30 consecutive images collected prior to P4 stimulation) and peak fluorescence intensity (mean of 4–5 consecutive images spanning the peak of the P4-induced $[Ca^{2+}]_i$ transient) were determined for each cell. P4-induced fluorescence increment was then calculated by subtracting resting fluorescence from peak fluorescence.
