# Supplementary material for: Single-cell analysis of [Ca2+]i signalling in sub-fertile men: characteristics and relation to fertilization outcome
Source: Hum Reprod. 2018 Apr 25;33(6):1023–33. doi: 10.1093/humrep/dey096 (PMC5972555; doi:10.1093/humrep/dey096)
Supplement: Supplementary Figure 3 [file dey096suppl_figure3.pdf]

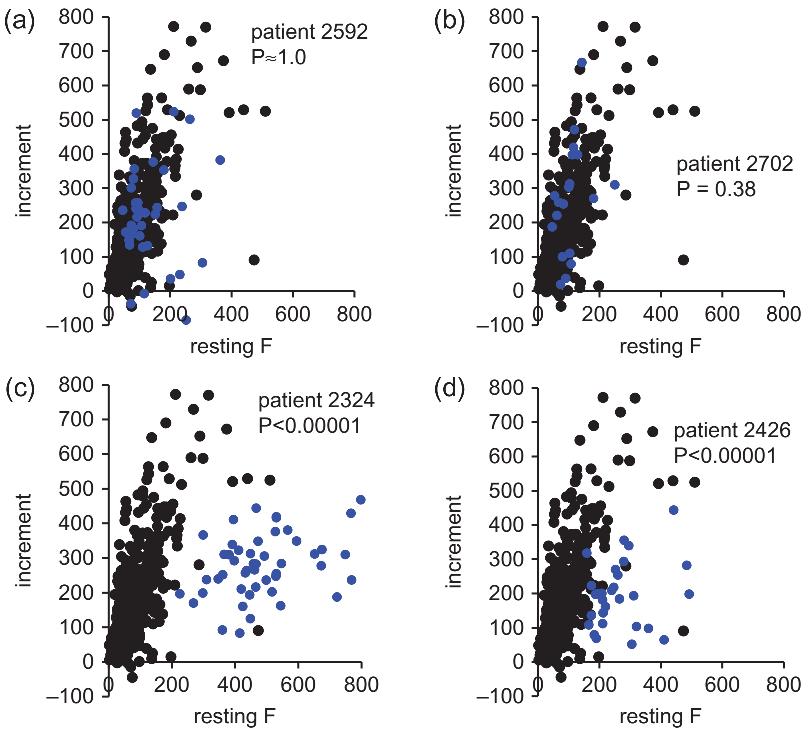

Supplementary Figure S3 Examples of relationship between mean resting fluorescence and mean fluorescence increment in four IVF(+ve) patients ((**a**–**d**) 39, 24, 53 and 30 cells, respectively; blue symbols). In each panel the black points show data from donor cells for comparison (749 cells from 21 donor samples). Numbers in each panel are patient code (for comparison with Fig. 1d) and *P* values show comparison of patient regression coefficient with that for donor cells.
