# Supplementary material for: Single-cell analysis of [Ca2+]i signalling in sub-fertile men: characteristics and relation to fertilization outcome
Source: Hum Reprod. 2018 Apr 25;33(6):1023–33. doi: 10.1093/humrep/dey096 (PMC5972555; doi:10.1093/humrep/dey096)
Supplement: Supplementary Figure 4 [file dey096suppl_figure4.pdf]

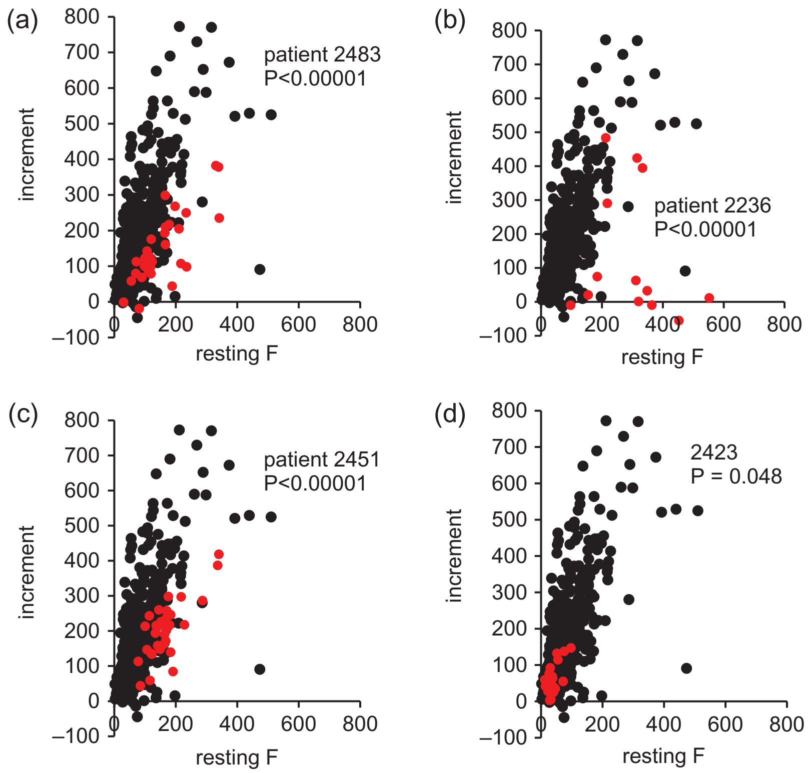

Supplementary Figure S4 Examples of relationship between mean resting fluorescence and mean fluorescence increment in four IVF-FF patients ((**a**–**d**) 34, 13, 33 and 23 cells, respectively; red symbols). In each of panel the black points show data from donor cells for comparison (749 cells from 21 donor samples). Numbers in each panel are patient code (for comparison with Fig. 1e) and *P* values show comparison of patient regression coefficient with that for donor cells.
