# Supplementary material for: Single-cell analysis of [Ca2+]i signalling in sub-fertile men: characteristics and relation to fertilization outcome
Source: Hum Reprod. 2018 Apr 25;33(6):1023–33. doi: 10.1093/humrep/dey096 (PMC5972555; doi:10.1093/humrep/dey096)
Supplement: Supplementary Figure 6 [file dey096suppl_figure6.pdf]

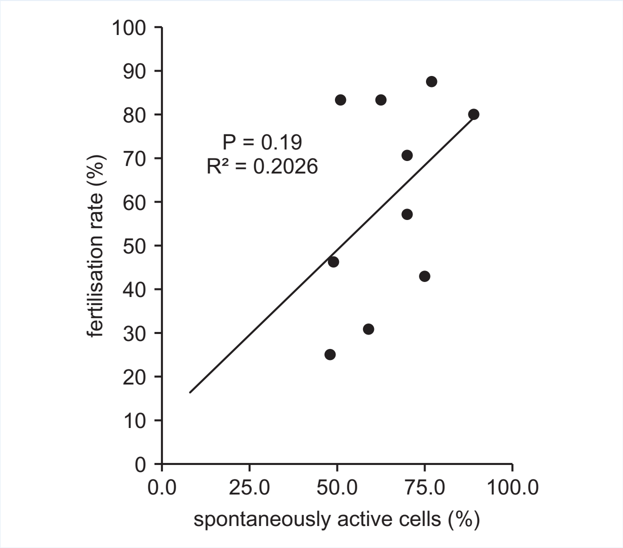

Supplementary Figure S6 Spontaneous $[Ca^{2+}]_i$ oscillations and IVF fertilization rate. Plot shows relationship between proportion of cells in which large spontaneous $[Ca^{2+}]_i$ oscillations were observed ($n = 10$ samples where such oscillations occurred) and the fertilization rate (%) achieved at IVF with that sample.
