# Supplementary material for: Single-cell analysis of [Ca2+]i signalling in sub-fertile men: characteristics and relation to fertilization outcome
Source: Hum Reprod. 2018 Apr 25;33(6):1023–33. doi: 10.1093/humrep/dey096 (PMC5972555; doi:10.1093/humrep/dey096)
Supplement: Supplementary Figure 7 [file dey096suppl_figure7.pdf]

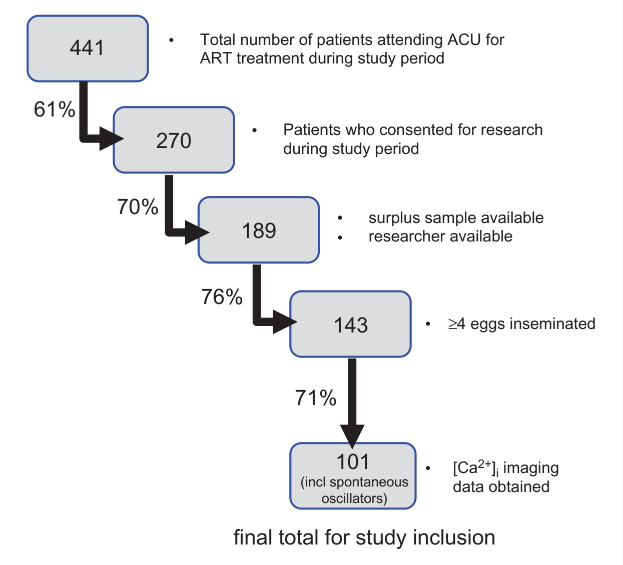

Supplementary Figure S7 Selection criteria for patient inclusion. A total of 441 couples attended the ACU for ART treatment during the study period, 270 of these (61%) were consented for research (donor sperm for female same sex couples was not included in study). For 189 (70%) of the consented couples there was adequate surplus sample for research and expertise was available on the day to carry out experiments. Only 143 of these 189 patients (76%) met the minimum four egg insemination criteria and of these there were 101 samples (71%) where we successfully obtained $[Ca^{2+}]_i$ imaging data.
